# Study of contaminants in natural cosmetics

**DOI:** 10.13075/ijomeh.1896.02617

**Published:** 2026

**Authors:** Agnieszka Rybka, Adam Sidor, Małgorzata Marć, Maria Kunysz, Dorota Szczepańska, Edyta Barnaś

**Affiliations:** 1 University of Rzeszow, Doctoral School, Collegium Medicum, Rzeszów, Poland; 2 Voivodeship Sanitary and Epidemiology Station in Rzeszow, Rzeszów, Poland; 3 University of Rzeszow, Faculty of Medicine, Medical College, Rzeszów, Poland; 4 University of Rzeszów, Faculty of Health Sciences and Psychology, Collegium Medicum, Rzeszów, Poland

**Keywords:** formaldehyde, heavy metals, *Staphylococcus aureus*, natural cosmetics, chemical and biological contaminants, aerobic mesophilic microorganisms

## Abstract

**Objectives:**

Cosmetic products placed on the market should be safe for both the consumer and the professional user. Safety monitoring of cosmetics is regulated by law in the European Union. In practice, there are 3 levels of safety assessment: the manufacturer, a qualified safety assessor and the competent authority, which in Poland is the State Sanitary Inspectorate, responsible also for official surveillance and product sampling. To identify the occurrence of threats to human health from selected chemical and biological factors based on cosmetic products distributed in Podkarpackie Province in 2023.

**Material and Methods:**

The material was 40 randomly selected samples of cosmetic products for body, face and hair. Formaldehyde was determined by spectrophotometry, silver(I) nitrate (AgNO_3_) by spectrophotometric methods, and mercury by atomic absorption spectrometry with an amalgamation technique. In addition, the total number of aerobic mesophilic microorganisms and *Staphylococcus aureus* were analyzed.

**Results:**

Formaldehyde content was <0.005% in all tested cosmetics for skin and hair cleansing. Mercury ranged from 0.0000001±0.00000001% to 0.0000002±0.00000003%. Silver(I) nitrate(V) in eye products was <1.57%. The total count of aerobic microorganisms was <10 CFU/g, and *Staphylococcus aureus* was not detected.

**Conclusions:**

Cosmetic products tested in 2023 showed formaldehyde below the detection limit (<0.005%), while mercury, AgNO_3_, and microbiological contaminants were within legal limits. Results apply only to the analyzed samples and should be interpreted considering current formaldehyde limits in cosmetics (0.001%, 10 ppm).

## Highlights

- The interest in natural products in cosmetology requires constant supervision.
- Chemical and biological testing reduces the risk of irritation and allergies.
- This encourages consumers to choose natural cosmetics.
- The selected cosmetics analyzed met European standards.

## INTRODUCTION

Cosmetic products marketed in the European Union (EU) are intended to be safe for those who use them. Already at the formulation stage, manufacturers should consider the restrictions on certain substances listed in Annex III of Regulation (EC) No. 1223/2009 of the European Parliament and of the Council of 30 November 2009 on cosmetic products (hereinafter Regulation (EC) No. 1223/2009) [1] and prohibited substances that should not be included in the product composition. Legislation clearly and precisely defines the rules for using: silver(I) nitrate(V) (AgNO_3_), formaldehyde and mercury in cosmetics [1]. This is of great importance to the people who use these products on a professional basis. Moreover, consumers are concerned about the presence of potent allergenic substances in cosmetic formulations. Such substances may be present due to inadequate risk management by the industry. These issues are addressed by the Scientific Committee on Consumer Safety (SCCS) in its Guidelines for the Evaluation of the Toxicological Documentation of Cosmetic Products [2]. A further undesired issue may be the risk of microbiological contamination of a cosmetic product, which increases with inappropriate hygiene habits, such as not washing hands before and after using a balm/cream, sharing cosmetics between consumers, using them after the expiry date or inappropriate storage [3]. Furthermore, the origin of cosmetics from different parts of the world, including those outside the EU, which do not always meet the requirements of Regulation (EC) No. 1223/2009, can be a problem. Inspections of cosmetic products, including laboratory testing of their representative samples, are carried out to verify compliance within the market [1]. The tasks of the State Sanitary Inspectorate related to public health include, i.e., monitoring cosmetic products [4]. Based on the internal guidelines of the Chief Sanitary Inspector dated December 22, 2022, for provincial sanitary and epidemiological stations in Poland [5], a sampling plan for microbiological and chemical testing of cosmetic products with declarations such as “natural,” “eco” and with reduced preservative content was developed in 2023 in Poland. It should be emphasized that in the case of chemical substances, it is not only a question of threats to human health, but also to the environment.

### Aims

Identifying the occurrence of chemical and biological hazards to human health, especially those with carcinogenic or mutagenic effects, based on the preliminary results of testing selected natural “eco” cosmetic products marketed in the Podkarpackie Province, Poland, in 2023.

## MATERIAL AND METHODS

The method used was laboratory measurement of specific cosmetic products. Formaldehyde and AgNO_3_ were determined based on the Regulation of the Minister of Health of March 19, 2020 on methods for determining samples necessary for the safety control of cosmetic products [6]. Formaldehyde was measured using a UV-1601 spectrophotometer (Shimadzu Corporation, Kyoto, Japan). The silver content was determined using a SOLAAR M6 MK2 spectrophotometer (Thermo Electron Corporation, Cambridge, United Kingdom) and then converted to the AgNO_3_ content in the sample. The method used for mercury (Hg) was absorption atomic spectrometry with amalgamation, performed on a DMA-80 mercury analyser (Milestone S.r.l., Sorisole, Italy). To determine the total number of mesophilic aerobic microorganisms (bacteria plus yeasts and moulds), a method based on the following standards was used: PN-EN ISO 21149:2017-07+A1: 2023-01 – Cosmetics – Microbiology – Enumeration and detection of mesophilic aerobic bacteria [7] and PN-EN ISO 16212:2017-08+A1: 2023-01 – Cosmetics – Microbiology – Enumeration of yeast and mould counts [8]. In the case of detecting the presence of *Staphylococcus aureus*, the PN-EN ISO 22718:2016-01+A1: 2023-01 – Cosmetics – Microbiology – Detection of *Staphylococcus aureus* was used [9]. The test results obtained were compared with the criteria set out in PN-EN ISO 17516:2014-11 Cosmetics – Microbiology – Limits for microbiological testing [10]. The conditions under which the tests were performed and the type of equipment used are specified in PN-EN ISO 21148:2017-07 – Cosmetics – Microbiology – General guidelines for microbiological examination [11] and the above-mentioned methodological standards.

The test consisted of 40 samples of cosmetic products intended for body, face and hair care, collected in 9 randomly selected districts. The laboratory tests included both chemical analysis (measurement of formaldehyde, mercury and AgNO_3_) and microbiological tests (number of mesophilic aerobic microorganisms and detection of *Staphylococcus aureus*). Detailed characteristics of the samples, including type, quantity, volume/weight, are presented in Table 1. One sample consisted of 2 packages of a cosmetic product from the same batch. Sampling, considering the above criteria, was carried out on a random basis.

**Table 1.** Characteristics of the cosmetic samples in the study on contaminants in natural cosmetic products for body, face and hair, Podkarpackie Province, Poland, 2023

Variable	Samples (N = 40) [n]	Type of cosmetics	Volume/Weight	County
Measurement				
formaldehyde	7	skin and hair cleansing products (hand soap, exfoliating shampoo, shower gel, bath fluid)	200 ml, 250 ml, 400 ml, 500 ml, 900 ml	Stalowa Wola
mercury (Hg)	7	colour cosmetics (eye shadows, mascaras) and facial care products (eye creams)	8.4 g, 10.8 g, 10 ml (eye shadows and mascaras), 15 ml (eye creams)	Łańcut
silver(I) nitrate(V) (AgNO_3_)_3_	6	eyebrow and eyelash henna, mascaras	7–8 ml, 15 ml, 15 g	4 samples: Rzeszów, 2 samples: Jarosław
Substance				
mesophilic aerobic microorganisms and *Staphylococcus aureus*	20	facial and body care products (facial milks, balms, facial and eye creams, facial and eye make-up removers)	15–400 ml (2 packages of the same batch: 15 ml, 30 ml, 50 ml, 100 ml – creams, 75 ml, 200 ml, 400 ml – milks/balms)	Dębica, Krosno, Przemyśl, Sanok, Rzeszów, Tarnobrzeg

The formaldehyde content in cosmetic samples was determined using a colorimetric method with pentane-2,4-dione, followed by measurement of the optical density of the extract at a wavelength of 410 nm. Approximately 1 g of a standardised sample of the tested cosmetic was taken for the determination. In order to determine the formaldehyde content, measurements were made using a standard curve covering the formaldehyde content (5–25 µg). To read the amount of formaldehyde from the standard curve, the value A2 was subtracted from A1.

The formaldehyde content in the sample (% m/m) was calculated using the following formula:

(1) $$\%(\text{m}/\text{m})\text{Formaldehyde}=\frac{\text{c}}{10^{3}\times \text{m}}$$

where:

c – amount of formaldehyde (µg) in the sample solution,
m – mass of the analytical sample (g).

The final result of the determination is the average of 2 parallel determinations. The range of the method was 0.005–0.025% (m/m). A water bath (60°C) and a non-automatic electronic balance AS 220.R2 PLUS (Radwag, Radom, Poland) were used for the analysis.

Mercury was determined in cosmetic samples using a DMA80 device by atomic absorption after thermal decomposition and amalgamation, without acid mineralisation. Approximately 100 mg of the analytical sample of the tested cosmetic is taken for a single determination.

The final result is the average of 2 readings. The method range was 0.001–1.00 mg/kg. The DMA-80 analyzer was operated at 0–35°C and relative humidity ≤80%. Samples were weighed using an XS 205 DU (Mettler-Toledo AG, Greifensee, Switzerland) electronic balance.

In order to determine the AgNO_3_ content, approx. 0.1 g of the sample was taken, dissolved in 0.02 M nitric acid(V) solution and the absorbance was measured. The silver content was read from the standard curve (1.0–5.0 µg/ml) and converted to AgNO_3_ (% m/m) according to the formula:

(2) $$\%(\text{m}/\text{m}){\text{AgNO}}_{3}=\frac{1.5748\times \text{c}}{10\times \text{m}}$$

where:

c – silver concentration in the sample solution (µg/ml),
m – sample weight (g).

The range of the AgNO_3_ determination method in mass percentage (% m/m) is 1.57–7.87%.

The final result is the average of 2 determinations. A non-automatic electronic balance WPS 720/C/2 (Radwag, Radom, Poland) was used for weighing.

Samples (microbiological contamination) were taken from cosmetic products with declared natural properties: 100% plant-based, eco-friendly, free from parabens and preservatives, or with reduced levels of these substances. A 1 g/ml sample in Eugon broth (Liofilchem, Roseto degli Abruzzi, Italy) was prepared for microbiological testing. Certified media were used: Sabouard with dextrose and chloramphenicol (SDCA) (Merck, Darmstadt, Germany), Baird-Parker (BP) (Oxoid, Basingstoke, UK), and tryptose soy agar (TSA) (Oxoid, Basingstoke, UK). Incubation was carried out at 32±2.5°C and 25±2.5°C. Neutralisation tests were performed in parallel to check the growth of microorganisms in the presence of a neutraliser. Strains from a recognised standardised collection were used for the tests. The equipment used included pipettes, glassware and incubators, which were monitored in accordance with the laboratory schedule. The results are presented in relation to the standards: number of mesophilic aerobic microorganisms in 1 g/ml and presence of *Staphylococcus aureus* (absent in 1 g).

The tests were carried out between May 8, 2023 and Ju-ne 30, 2023.

## RESULTS

The determination of formaldehyde in cosmetics intended for washing the skin and hair, including hand soap, exfoliating shampoo, shower gel, and bath fluid, showed results <0.005% in all tested samples. These results presented in Table 2.

**Table 2.** Determination of formaldehyde content in the cosmetic samples taken in Stalowa Wola, Poland, 2023

Bath product sample	Formaldehyde [%]
Sample 1: bath liquid (900 ml)	<0.005
Sample 2: shower gel (500 ml)	<0.005
Sample 3: hair shampoo (250 ml)	<0.005
Sample 4: bath liquid (400 ml)	<0.005
Sample 5: shower gel (400 ml)	<0.005
Sample 6: exfoliating shampoo (200 ml)	<0.005
Sample 7: hand soap (500 ml)	<0.005

1 sample = 2 packages of the product from the same batch.

Mercury content in eye makeup products, such as eyeshadow palettes and eye creams, ranged from 0.0000001±0.00000001% to 0.0000002±0.00000003%. These results are presented in Table 3. In the case of samples No. 1 and No. 7, 9 subsamples (1a–i) and 6 subsamples (7a–f) were analyzed, respectively, which came from a single product belonging to the same production batch. The subsamples were analyzed separately. However, for the purposes of counting and analyzing the samples, they were treated as a single sample.

**Table 3.** Determination of mercury content in the collected cosmetic samples in Łańcut, Poland, 2023

Sample	Mass	Mercury	
		mg/kg	% (g/100 g)
Sample 1: Eyeshadow palette			
colour 1 (a)	10.8 g	0.001±0.0001	0.0000001±0.00000001
colour 2 (b)	10.8 g	0.002±0.0003	0.0000002±0.00000003
colour 3 (c)	10.8 g	0.001±0.0001	0.0000001±0.00000001
colour 4 (d)	10.8 g	0.001±0.0001	0.0000001±0.00000001
colour 5 (e)	10.8 g	0.001±0.0001	0.0000001±0.00000001
colour 6 (f)	10.8 g	<0.001	<0.0000001
colour 7 (g)	10.8 g	<0.001	<0.0000001
colour 8 (h)	10.8 g	<0.001	<0.0000001
colour 9 (i)	10.8 g	0.002±0.0003	0.0000002±0.00000003
Sample 2: eye cream I product	15 ml	<0.001	<0.0000001
Sample 3: eye cream II product	15 ml	<0.001	<0.0000001
Sample 4: eye cream III product	15 ml	<0.001	<0.0000001
Sample 5: mascara product I	10 ml	<0.001	<0.0000001
Sample 6: mascara product II	10 ml	<0.001	<0.0000001
Sample 7: eye shadow palette		<0.001	<0.0000001
glossy heather colour (a)	8.4 g		
glossy brick colour (b)	8.4 g		
matte heather colour (c)	8.4 g		
apricot colour (d)	8.4 g		
beige colour (e)	8.4 g		
brown colour (f)	8.4 g		

Samples No. 1 (sub-samples a-i) and 7 (sub-samples a–f) consist of sub-samples taken from a single product from the same production batch and were treated as a single sample (N = 1) in the analysis.

1 sample = 2 packages of the product from the same batch.

The content of AgNO_3_ in cosmetics, including mascara and henna for eyebrows and eyelashes, was below 1.57% in all tested samples. These results presented in Table 4.

**Table 4.** Determination of silver nitrate (AgNO_3_) content in the collected cosmetic samples, Poland, 2023

Sample	AgNO_3_ [%]	County
Sample 1: I product – eyebrow henna (15 ml)	<1.57	Rzeszów
Sample 2: II product – eyebrow henna (15 g)	<1.57	Rzeszów
Sample 3: III product – eyebrow and eyelash henna (15 ml)	<1.57	Rzeszów
Sample 4: IV product – eyebrow and eyelash henna (15 ml)	<1.57	Rzeszów
Sample 5: V product – mascara (8 ml)	<1.57	Jarosław
Sample 6: VI product – mascara (7 ml)	<1.57	Jarosław

1 sample = 2 packages of the product from the same batch.

The analysis showed that the limit for aerobic mesophilic microorganisms (bacteria plus yeasts and molds) was not exceeded. The result was below the detection limit (no microbial growth on plates with a dilution of 10^1^, 10^2^). In addition, no *Staphylococcus aureus* bacteria were found in the tested sample. These results presented in Table 5.

**Table 5.** Determination of the content of mesophilic aerobic microorganisms and *Staphylococcus aureus* in the collected cosmetic samples, Poland, 2023

Sample	Mesophilic aerobic microorganisms^a^ [CFU/g]	*Staphylococcus aureus* in 1 g	Method		County
			mesophilic microorganisms	*Staphylococcus aureus*	
Sample 1: face cream (50 ml)	<10	absent	1, 2	3	Dębica
Sample 2: body lotion (400 ml)	<10	absent	1, 2	3	Dębica
Sample 3: eye cream (30 ml)	<10	absent	1, 2	3	Dębica
Sample 4: face cream (50 ml)	<10	absent	1, 2	3	Krosno
Sample 5: face cream (50 ml)	<10	absent	1, 2	3	Krosno
Sample 6: face cream (50 ml)	<10	absent	1, 2	3	Krosno
Sample 7: face cream (50 ml)	<10	absent	1, 2	3	Przemyśl
Sample 8: face cream (50 ml)	<10	absent	1, 2	3	Przemyśl
Sample 9: face cream (50 ml)	<10	absent	1, 2	3	Przemyśl
Sample 10: face cream (50 ml)	<10	absent	1, 2	3	Rzeszów
Sample 11: face cream (50 ml)	<10	absent	1, 2	3	Rzeszów
Sample 12: body milk (400 ml)	<10	absent	1, 2	3	Rzeszów
Sample 13: body lotion (400 ml)	<10	absent	1, 2	3	Rzeszów
Sample 14: body lotion (200 ml)	<10	absent	1, 2	3	Sanok
Sample 15: face cream (50 ml)	<10	absent	1, 2	3	Sanok
Sample 16: body lotion (75 ml)	<10	absent	1, 2	3	Sanok
Sample 17: eye cream (15 ml)	<10	absent	1, 2	3	Tarnobrzeg
Sample 18: body milk (400 ml)	<10	absent	1, 2	3	Tarnobrzeg
Sample 19: face and eye makeup remover milk (200 ml)	<10	absent	1, 2	3	Tarnobrzeg
Sample 20: face cream (100 ml)	<10	absent	1, 2	3	Tarnobrzeg

Method 1 – PN-EN ISO 21149:2017-07 [7]; method 2 – PN-EN ISO 16212:2017-08 [8]; method 3 – PN-EN ISO 22718:2016-01 [9].

a Bacteria plus yeasts and molds.

## DISCUSSION

### Formaldehyde

In the pilot study, 1 of the substances tested was formaldehyde, which is classified by the International Agency for Research on Cancer (IARC) as a group 1 human carcinogen. However, a review of 21 epidemiological studies from the period 2000–2021 shows that exposure is weakly associated with lung cancer, nasopharyngeal cancer, leukaemia, and non-Hodgkin lymphoma [12]. Research conducted in Taiwan among 414 employees, comparing them with 172 people in the control group, showed that even low doses of formaldehyde can lead to health problems. This mainly concerned allergic rhinitis and atopic dermatitis, as well as other irritations. These effects were particularly noticeable in people working in occupations with higher exposure, such as anatomical pathology [13]. It is also worth mentioning that formaldehyde is classified in accordance with Regulation (EC) No. 1272/2008 of the European Parliament and of the Council of December 16, 2008, on classification, labeling, and packaging of substances and mixtures [14] (hereinafter referred to as the CLP Regulation) as a category 1B carcinogen (Carc. 1B) and as a skin sensitizer category 1A (Skin Sens. 1A).

The formaldehyde content was <0.005% in cosmetics intended for washing the skin and cleansing the hair (hand soap, exfoliating shampoo, shower gel, bath gel), which means that it may >0.001%. If the formaldehyde concentration in finished products exceeds 0.001% (regardless of the amount of formaldehyde-releasing substances in the finished product), it should be indicated on the label [1,6].

However, it is necessary to take into account the provision of recital 2 of Annex V (concerning the list of preservatives in cosmetic products) of Regulation (EC) No. 1223/2009 [1], which states that a ready-made product containing formaldehyde in such a concentration may be placed on the market in the EU until 31 July 2024 and made available on the EU market until 31 July 2026. This means that the formaldehyde result shown in Table 2 in samples taken in 2023 is acceptable and does not need to be reported on the label.

The method described in the methodology enabled the determination of formaldehyde above the limit of quantification, but did not allow for the detection of lower, regulatory-relevant levels. Therefore, the results should be interpreted solely as information about the presence of formaldehyde above the sensitivity of the method used. At the same time, the method used is characterised by high repeatability, stability of results and simplicity of execution, and the sample weight of approx. 1 g allowed for easy dissolution and obtaining a homogeneous solution from a single sample. In future studies, it is therefore reasonable to use more sensitive analytical methods in order to obtain more accurate results.

Based on a review of the RAPEX database aimed at finding products with high concentrations of preservatives and microbiological contamination, Neza and Centini [15] observed that 32 hair care products were recalled due to high concentrations of formaldehyde (0.3–25%). However, it should be noted that the majority of these products came from outside the EU (Brazil, USA, England, China or unknown origin). The high concentration of formaldehyde is probably due to the unintentional import of these cosmetics into the EU. The results from Spain are worrying, where the concentration in a hair product stabilizer was 25%.

It should be noted that formaldehyde acts as a preservative in cosmetics due to its antimicrobial effect, especially at high concentrations. Low concentrations may lead to microbiological contamination of the cosmetic. A negative effect of formaldehyde in cosmetic products is its toxic effect on the consumer [16].

Awareness of exposure to harmful chemicals concerns not only consumers but also workers in the beauty industry. The results of a study by Asare-Donkor et al. [17] seem very interesting in this regard. The authors investigated the level of knowledge of employees in 60 beauty salons about the products they use at work, including hair, nails, etc, showing that the subjects suffered from respiratory, nasal, eye and skin irritation, as well as mild headaches. This may be caused by formaldehyde coming into contact with the skin. All workers felt that their health was affected by formaldehyde exposure. Only 2% of the hairdressers surveyed knew the chemical composition of the products they used.

In a study conducted in California by Johnson et al. [18] among women of different skin phenotypes (Black, Vietnamese and Hispanic) using personal care products (i.e., intimate hygiene, hair, makeup, skin, nails and perfume/deodorant products), the presence of ≤65% of the ingredients listed on the label was found to be impurities. The data for 74% of undisclosed ingredients, e.g., preservatives and those under the name “fragrance” are worrying. Formaldehyde was the most common chemical found. The authors claim that full disclosure of ingredients, combined with increased awareness of the risks of harmful chemicals, would significantly influence consumers’ purchasing decisions in favor of products that are safe for their health. Other authors also emphasize the need for full labeling of the health effects of chemicals in products to increase the likelihood of informed use [18–21].

It should be emphasized that misleading the consumer by failing to indicate intentionally added ingredients on the containers and outer packaging of cosmetic products is contrary to Article 19(1)(g) of Regulation (EC) No. 1223/2009 on cosmetic products [1].

### Mercury

Another substance tested in cosmetics was mercury. After inhaling mercury or exposure to it through the skin, health symptoms such as neurological and behavioral disorders appear. In addition, mercury, especially in the form of methylmercury, is considered a neurotoxic and reprotoxic substance, with a documented effect on the nervous system and fetal development [22].

In this study, the mercury concentration in mg/kg, converted to % (g/100 g), was between 0.0000001±0.00000001% and 0.0000002±0.00000003%. According to Annex III of Regulation (EC) No. 1223/2009 [1] concerning the list of substances which may be contained in cosmetic products subject to specific restrictions for cosmetic products, the maximum concentration of thiomersal and phenylmercury salts including borates in the ready-to-use preparation for mercury is 0.007%. The obtained result indicates that the concentration of this ingredient in cosmetic products intended for the eyes (eyeliner, mascara, eye cream) is not exceeded.

Based on a systematic review by Bastianasz et al. [23] of mercury concentrations in samples of skin whitening products (e.g., creams), it was concluded that there were no significant differences in changes in mercury concentrations over a 22-year sampling period. Based on 25 studies of 552 creams, including 36 facial creams, the overall median central mercury concentration was 0.49 µg/g. The highest amount was found in creams (314.387 µg/g), including facial creams (35.824 µg/g). In contrast, the highest median concentration was found in products from the WHO-affiliated countries of the Eastern Mediterranean, Southeast Asia and the Western Pacific. It is worth noting that these results are higher than those obtained in the authors’ study and listed in Table 3 (0.1±0.01 µg to 0.2±0.03 µg).

Bastianasz et al. found a variable frequency of complaints related to mercury exposure in the group of 832 identified individuals with the most common recurrent complaints such as headaches and depression. In Jamaica, these were found in only 31 people, but in the USA and Hong Kong, as many as 268 people were affected.

### Silver(I) nitrate(V)

Silver(I) nitrate(V) is a substance which, pursuant to the CLP Regulation, has been classified as a hazardous substance causing skin burns and eye damage [14]. In addition, it is classified by many suppliers in the European Chemicals Agency (ECHA) C&L Inventory as a substance that may be harmful to reproduction (Repr. 1B/H360D) [24]. Health risks resulting from exposure to soluble forms of silver also include argyria and argyrosis, i.e., permanent bluish-gray discoloration of the skin and eyes. Other toxic effects include irritation of the eyes, skin, gastrointestinal tract, and respiratory tract, damage to the kidneys and liver, and changes in blood cells [25].

In the analysis of AgNO_3_, the result was <1.57%. According to Annex III of Regulation (EC) No. 1223/2009 [1] on the list of substances that may be included in cosmetic products subject to certain restrictions, the maximum concentration of AgNO_3_ in the ready-to-use preparation is 4%. However, it should be noted that, according to this Annex, its use is permitted only for dyeing eyebrows and eyelashes. The obtained result indicates that the concentration of this ingredient is not exceeded in cosmetic products intended as colorants (mascara and henna for eyebrows and eyelashes). The available literature on AgNO_3_ does not provide information on concentration results in samples of cosmetic products. However, the importance of silver nanoparticles in the cosmetic industry is highlighted in products such as eye shadows. Silver nanoparticles with a size of 7 nm were found to be most effective against the bacterium *Staphylococcus aureus* [26].

### Microbiological contamination

Mesophilic aerobic microorganisms (bacteria, yeasts, and molds) were not detected. Acording to PN-EN ISO 16212:2017-08+A1:2023-01 and PN-EN ISO 21149:2017-07+A1:2023-01, the result indicates no growth of countable microorganisms on plates with dilutions from 10^–1^ to 10^–2^ [7,8]. A value <10 CFU/g for the number of mesophilic aerobic microorganisms (bacteria plus yeasts and molds) and the absence of *Staphylococcus aureus* bacteria in 1 g of products indicates that the maximum acceptable limit for the total number of aerobic microorganisms and specific microorganisms such as *Staphylococcus aureus* for category II cosmetics, for which the limit is 2.0 × 10^3^ CFU/g according to the PN-EN ISO 17516:2014-11 standard, according to which quantitative and qualitative tests are performed [10].

The analysis carried out by Alshehrei [27] in Saudi Arabia on 32 samples of personal care products showed that 2 samples (face and body cream) were not contaminated with *Staphylococcus aureus*. On the second day of incubation, contamination with this bacterium was observed at the level of 6.80 × 10^5^ CFU/ml. Therefore, the author recommends the use of preservatives to prevent contamination during storage and transport. In all incubation periods analyzed, the number of microorganisms (mesophilic bacteria and fungi) found was <10.

The results obtained by the authors, i.e., <10 mesophilic bacteria plus yeasts and molds, no contamination with *Staphylococcus aureus*, indicate the correct application of the preservative system in the “eco” samples, i.e., with a reduced content of preservatives. When comparing the authors’ results with Alshehrei's study [27], it is worth noting the regionalization of the samples: temperate climate (Poland) and desert, tropical and continental climate (Saudi Arabia). In addition, it is not known whether these were natural personal care products, as in the case of the samples taken in southern Poland.

The RAPEX review [15] shows that 62 products, including eye creams, skin care products, baby creams and lotions, were withdrawn due to contamination with mesophilic aerobic microorganisms (including bacteria, molds, yeasts) and *Staphylococcus aureus*, which was found in shea butter and massage cream.

In a study conducted by Almukainzi et al. [28] in Saudi Arabia on 21 products of different brands, including lip balms and hair creams, microbiological contamination was found in 14 cosmetic products at levels ranging 1471.5–299.5 CFU/ml. The highest level of contamination was found in baby oil – 1471.5 CFU/ml. *Staphylococcus aureus* was identified in 12 cosmetic samples: lip balm, make-up foam, creams, soap and deodorant. This is probably due to improper storage.

Finally, the lack of a clear definition of natural cosmetics as opposed to “eco” cosmetics can lead to consumer confusion about their composition and the identification of these terms as synonymous. In practice, however, organic cosmetics must contain at least 95% of ingredients from certified crops. They must not contain artificial colours or fragrances. Providing full information on the composition of selected cosmetic products, including the risk of harmful chemical substances, including carcinogens or mutagens and biological effects, is extremely important, especially in the current era of growing consumer health and environmental awareness.

## CONCLUSIONS

Preliminary analyses of cosmetic products tested in 2023, including liquid soaps, bath liquids, shower gels, and hair shampoos, showed formaldehyde levels below the detection limit (<0.005%). Mercury, AgNO_3_, and microbiological contaminants were not detected above current legal limits. These findings apply only to the analyzed samples, and results should be interpreted considering evolving regulatory limits for formaldehyde in cosmetics (labeling threshold now 0.001%, 10 ppm). Further research on a larger number of products and a broader range of substances is recommended.

## References

[1] Regulation (EC) No. 1223/2009 of the European Parliament and of the Council of November 30, 2009 on cosmetic products. Off J Eur Union. L 342, 22.12.2009, p. 59, as amended.

[2] Bialas I, Zelent-Kraciuk S, Jurowski K. The Skin sensitisation of cosmetic ingredients: Review of actual regulatory status. Toxics. 2023;11(4):392, 10.3390/toxics11040392. 37112619 PMC10146005

[3] Giorgio A, Miele L, De Bonis S, Conforti I, Palmiero L, Guida M, et al. Microbiological stability of cosmetics by using challenge test procedure. J Pure Appl Microbiol. 2018;12(1):23–8, 10.22207/JPAM.12.1.04.

[4] Act of March 14, 1985 on the State Sanitary Inspection. J Laws. 2024, item 416.

[5] Chief Sanitary Inspector. Internal guidelines on supervision of cosmetic products. Letter of 22 Dec 2022, CH.NR.45.100.2022. Warsaw: Chief Sanitary Inspector; 2022.

[6] Regulation of the Minister of Health of March 19, 2020, on the methods of determining the samples necessary for the safety control of cosmetic products. J Laws. 2020, item 931.

[7] PN-EN ISO 21149: 2017-07+A1: 2023-01 – Cosmetics – Microbiology – Enumeration and detection of aerobic mesophilic bacteria. Polish Committee for Standardization: Warsaw; 2023.

[8] PN-EN ISO 16212: 2017-08+A1: 2023-01 – Cosmetics – Microbiology – Enumeration of yeast and mould counts. Polish Committee for Standardization: Warsaw; 2023

[9] PN-EN ISO 22718: 2016-01+A1: 2023-01. Cosmetics – Microbiology – Detection of Staphylococcus aureus. Warsaw: Polish Committee for Standardization; 2023.

[10] PN-EN ISO 17516: 2014-11 Cosmetics – Microbiology – Microbiological limits. Warsaw: Polish Committee for Standardization; 2014.

[11] PN-EN ISO 21148: 2017-07. Cosmetics – Microbiology – General guidelines for microbiological examination. Warsaw: Polish Committee for Standardization; 2017.

[12] Protano C, Buomprisco G, Cammalleri V, Pocino RN, Marotta D, Simonazzi S, et al. The carcinogenic effects of formaldehyde occupational exposure: a systematic review. Cancers (Basel). 2021;14(1):165, 10.3390/cancers14010165. 35008329 PMC8749969

[13] Fan HY, Lin JP, Yang TA, Tsao YC. Health effects of low-dose formaldehyde exposure: a cross-sectional study in occupational settings. Int J Occup Med Environ Health. 2025;38(3):236–48, 10.13075/ijomeh.1896.02503. 40511596 PMC12273900

[14] Regulation (EC) No 1272/2008 of the European Parliament and of the Council of 16 December 2008 on classification, labeling and packaging of substances and mixtures, amending and repealing Directives 67/548/EEC and 1999/45/EC, and amending Regulation (EC) No 1907/2006. Off J Eur Union. L 353, 31.12.2008, pp. 1–1355, as amended.

[15] Neza E, Centini M. Microbiologically contaminated and over-preserved cosmetic products according Rapex 2008–2014. Cosmetics. 2016;3(1):3, 10.3390/cosmetics3010003.

[16] Halla N, Fernandes IP, Heleno SA, Costa P, Boucherit-Otmani Z, Boucherit K, et al. Cosmetics preservation: A review on present strategies. Molecules. 2018;28;23(7):1571, 10.3390/molecules23071571. 29958439 PMC6099538

[17] Asare-Donkor NK, Kusi Appiah J, Torve V, Voegborlo RB, Adimado AA. formaldehyde exposure and its potential health risk in some beauty salons in Kumasi metropolis. J Toxicol. 2020;2020:88751678875167, 10.1155/2020/8875167. PMC7661109 33204257

[18] Johnson PI, Favela K, Jarin J, Le AM, Clark PY, Fu L, et al. Chemicals of concern in personal care products used by women of color in three communities of California. J Expo Sci Environ Epidemiol 2022;32:864–76, 10.1038/s41370-022-00485-y. 36323919 PMC9628299

[19] Bilal M, Mehmood S, Iqbal HMN. The Beast of beauty: environmental and health concerns of toxic components in cosmetics. Cosmetics 2020;7(1):13, 10.3390/cosmetics7010013.

[20] Helm JS, Nishioka M, Brody JG, Rudel RA, Dodson RE. Measurement of endocrine disrupting and asthma-associated chemicals in hair products used by Black women. Environ Res. 2018;1(165):448–58, 10.1016/j.envres.2018.03.030. 29705122

[21] Alnuqaydan AM. The dark side of beauty: an in-depth analysis of the health hazards and toxicological impact of synthetic cosmetics and personal care products. Front Public Health. 2024;12:1439027, 10.3389/fpubh.2024.1439027. 39253281 PMC11381309

[22] World Health Organization (WHO). Mercury and health [Internet]. Geneva: WHO; 2024 [cited 2026 Mar 26]. Available from: https://www.who.int/news-room/fact-sheets/detail/mercury-and-health

[23] Bastiansz A, Ewald J, Saldaña VR, Santa-Rios A, Basu N. A Systematic Review of Mercury Exposures from Skin-Lightening Products. Environ Health Perspect. 2022;130(11):116002, 10.1289/EHP10808. 36367779 PMC9651181

[24] European Chemicals Agency (ECHA). Silver nitrate – Substance Information [Internet]. Helsinki: ECHA [cited 2025 Dec 26]. Available from: https://echa.europa.eu/substance-information/-/substanceinfo/100.028.958.

[25] Drake PL, Hazelwood KJ. Exposure-related health effects of silver and silver compounds: a review. Ann Occup Hyg. 2005;49(7):575–85 10.1093/annhyg/mei019. 15964881

[26] Ong WTJ, Nyam KL. Evaluation of silver nanoparticles in cosmeceutical and potential biosafety complications. Saudi J Biol Sci. 2022;29(4):2085–94, 10.1016/j.sjbs.2022.01.035. 35531241 PMC9073040

[27] Alshehrei FM. Microbiological quality assessment of skin and body care cosmetics by using challenge test. Saudi J Biol Sci. 2024;31(4):103965, 10.1016/j.sjbs.2024.103965. 38440744 PMC10910155

[28] Almukainzi M, Alotaibi L, Abdulwahab A, Albukhary N, El Mahdy AM. Quality and safety investigation of commonly used topical cosmetic preparations. Sci Rep. 2022;12(1):18299, 10.1038/s41598-022-21771-7. 36316522 PMC9622732

